# Strategies for single base gene editing in an immortalized human cell line by CRISPR/Cas9 technology

**DOI:** 10.1007/s13205-023-03878-4

**Published:** 2024-01-19

**Authors:** Alda Corrado, Romina Aceto, Simona Miglietta, Roberto Silvestri, Irene Dell’Anno, Irene Lepori, Benedetta Ricci, Cristina Romei, Roberto Giovannoni, Laura Poliseno, Monica Evangelista, Marianna Vitiello, Monica Cipollini, Rossella Elisei, Stefano Landi, Federica Gemignani

**Affiliations:** 1 Department of Biology, Genetic Unit, University of Pisa, Via Derna 1, 56126 Pisa, Italy; 2 Humanitas Clinical and Research Centre- IRCCS, Via Manzoni 56, 20089 Milan, Italy; 3 San Raffaele Telethon Institute for Gene Therapy (SR-Tiget), IRCCS San Raffaele Scientific Institute, Via Olgettina 60, 20132 Milan, Italy; 4 Institute of Clinical Physiology (IFC), CNR, Via Giuseppe Moruzzi 1, 56124 Pisa, Italy; 5 Fondazione I.R.C.C.S., Istituto Neurologico Carlo Besta, Via Celoria 11, 20133 Milan, Italy; 6 Endocrine Unit, Department of Clinical and Experimental Medicine, University of Pisa, Via Paradisa 2, 56124 Pisa, Italy

**Keywords:** CRISPR/Cas9, Knock-in, Human cell lines, Single base DNA editing, Double nickase strategy

## Abstract

The use of CRISPR/Cas9 system has rapidly grown in the last years. Here, the optimization of gene editing of a single-nucleotide polymorphism in a human non-malignant somatic cell line of thyrocytes (Nthy-Ori) was described highlighting strategies for overcoming the problems concerning the delivery and off-targets. We employed both lentivirus and chemical lipids as delivery agents and two strategies for creating the double-strand breaks (DSB). The former induced a DSB by a classical Cas9 nuclease (standard strategy), while the second one employed a modified Cas9 creating a single-strand break (SSB). The knock-in was carried out using a single-stranded donor oligonucleotide or the HR410-PA donor vector (HR). The desired cells could be obtained by combining the double nickase system with the HR vector transfected chemically. This result could be due to the type of DSB, likely processed mainly by non-homologous end joining when blunt (standard strategy) and by HR when overhanging (double nickase). Our results showed that the double nickase is suitable for knocking-in the immortalized Nthy-Ori cell line, while the standard CRISPR/Cas9 system is suitable for gene knock-out creating in/del mutations.

**Supplementary Information:**

The online version contains supplementary material available at 10.1007/s13205-023-03878-4.

## Introduction

Nowadays, the clustered regularly interspaced short palindromic repeats/associated protein-9 nuclease (CRISPR/Cas9) system appears as the most promising technique of gene editing (Mali et al. 2013). It uses a nuclease guided by a short guide 20-nucleotide-long RNA (sgRNA) to target DNA through Watson–Crick pairing (Ran et al. 2013a). Cas9 nuclease is used for genome editing by formation of a double-strand break (DBS) at the target locus (Ran et al. 2013a). Then, the endogenous homologous recombination (HR) or the non-homologous end joining (NHEJ) pathways are exploited to obtain the desired modification. However, the delivery of Cas9 showed to be not always efficient, in particular depending upon the cell types employed (Walsh and Hochedlinger 2013). Another well-known hurdle is represented by the off-target effects due to the frequent occurrence of the protospacer adjacent motif (PAM) or to tolerated mispairings between the sgRNA and unspecific targets (Jiang and Doudna 2015; Segal and Meckler 2013). This work will describe the use of CRISPR/Cas9 for editing a single-nucleotide polymorphism (SNP) in a permanent human non-malignant cell line of thyrocytes, namely Nthy-Ori, highlighting the strategies for an increased efficiency. In particular, we evaluated (i) lentivirus or chemical lipids as delivery agents, (ii) classical or double nickase strategies (Ran et al. 2013b) for inducing the DSB, and (iii) single-strand donor oligonucleotide (ssODN) or HR vector for carrying the alternative allele. A special remark will be focused on the difficulty to obtain colonies from permanent non-malignant cell lines grown from single-cell progenitors, a fact that hampers the possibility to easily select the cells correctly gene edited.

## Material and methods

### Target sequence and cell lines

SV40-immortalized Nthy-Ori cells (Sigma-Aldrich, MO, USA) were employed as the model of non-malignant thyrocytes and they were subjected to modification of their normal C/A-heterozygote genotype in the polymorphic site rs4644 within the *LGALS3* gene. Cells were grown in RPMI1640 medium supplemented with 10% fetal bovine serum (FBS; EuroClone SpA, Milan, Italy). The human embryonic kidney (HEK) A293T (ATCC Manassas, VA, USA) cells were cultured in low-glucose DMEM supplemented with 10% FBS, 100 U/ml of penicillin, and 100 U/ml of streptomycin. All cultures were maintained at 37 °C in a 5%-CO_2_ humidified air.

### Delivery: chemical agents and lentivirus

Firstly, we compared Attractene (Qiagen, Hilden, Germany) vs Lipofectamine 3000 (Thermo Fisher Scientific, Waltham, MA, USA) by transfecting Nthy-Ori cells with AAY-3 green fluorescent protein (GFP) expression vector according to the manufacturer protocol. After 48 h post-transfection, cells were sorted by fluorescent-activated cell sorter (FACS; BD FACSJazz™; BD Biosciences, San Josè, CA, USA) for assessing the rate of transfection efficiency and cell viability.

Lentivector particles were generated by using the vectors psPAX2 for virus packaging, pMD2.G (Gag, Pol, Rev, and Tat), and for virus envelope and the vector genome carrying the gene of interest (lentivirus vector). They were co-transfected into HEK A293T cell lines by the use of PEI buffer according to the manufacturer’s protocol. After 48 h of incubation, the supernatant is collected and centrifuged to concentrate viral particles using the protocol proposed by Lenti-X Concentrator (Clontech, Takara). Target cells were transducted by polybrene agent, and, after 24 h post-plating, treated with puromycin (1 μg/ml) to select the transducted cells. The stable pCW-Cas9 cell line was transducted with the lentiviral pLX-sgRNA vector and the evaluation of the transduction was carried out by the co-treatment of cells with blasticidin (10 μg/ml) and puromycin: only cells correctly transducted (i.e., carrying the two vectors) were able to proliferate in the presence of both the antibiotics.

### CRISPR/Cas9 strategies

To determine the best approach for the knock-in, we evaluated three CRISPR/Cas9 strategies: standard, with lentivirus, and with double nickase. The workflows are summarized in Fig. S1.

(i) **Standard strategy.** We used all-in-one vector PX459 (pSpCas9(BB)-2A-Puro V2.0, gift from Feng Zhang (Addgene plasmid # 62988) (Ran et al. 2013b) having a puromycin resistance and native Cas9 (Fig. S2 panel A). The vector was assembled as described in Ran et al. (2013b). The presence of the sgRNA was detected by colony PCR and further verified by Sanger sequencing. To test the function of sgRNAs, Surveyor assay (Integrated DNA Technologies, IDT, Coralville, Iowa, USA) was performed following the protocol proposed by Ran et al. (2013b).
(ii) **Double nickase.** We employed two distinct all-in-one Cas9 mutated vectors (pSpCas9n(BB) (PX460), kindly donated by Feng Zhang (Addgene plasmid # 48873), each targeting adjacent regions of the interest locus (Fig. S2 panel B). The assembly and the sgRNA validation followed the protocol proposed by Ran et al. (2013b). The design of the best sgRNAs was carried out with CHOPCHOP (http://chopchop.cbu.uib.no/) and, for the double nickase strategy, ATUM (https://www.atum.bio/eCommerce/cas9/input).
(iii) **Lentivirus.** We employed pCw-Cas9 (Addgene plasmid # 50661) for the expression of the endonuclease-doxycycline-inducible Cas9 (Fig. S2 panel C) and pLX-sgRNA (Addgene plasmid # 50662), for inserting the custom sgRNA (Fig. S2 panel D). Both (kind gifts from Eric Lander and David Sabatini (Wang et al. 2014)) have an antibiotic resistance cassette, puromycin and blasticidin, respectively. Thus, the correct transduced cells were resistant to both antibiotics, abolishing the problem related to the delivery efficiency (Fig. S2 panel C and D). The best dosage of doxycycline (1 μg/ml) useful for the induction of Cas9 was evaluated with real-time PCR (RT-PCR) by measuring the Cas9 gene expression (Vitiello et al. 2017) and by screening three different doses 1, 2 and 4 μg/ml. sgRNAs were cloned into pLX-sgRNA by Overlap PCR replacing the AAVS1-targeting sgRNA present into the vector. The selection of the correct cloning was performed by colony PCR and validated by Sanger sequencing. All primers are listed in Table S1.

### Knock-in strategies: single-strand DNA oligonucleotide (ssODN) or donor vector

A critical point to perform a knock-in is the choice of the donor system. We tested two approaches: ssODN and a donor vector.

(i) **ssODN.** The synthetic ssODN was a 157-nucleotide-long single-strand DNA carrying the alleles A or C at the position 40 (Fig. S3) Moreover, it included three additional mutations designed to not affect the aminoacidic sequence, but useful for both masking other PAM sequences and creating a sequence-specific site for PCR selection of the correctly gene-edited clones.
(ii) **HR vector. **The donor vector, HR410-PA (HR vector; System Biosciences, SBI, Palo Alto, CA, USA), was designed to increase the HR rate. It has three important features: dual selection markers placed between the two multiple cloning sites (MCS), two insulators, and LoxP sites. Concerning the selection markers, the enrichment of HR-modified cells could be carried out either by cell sorting using the expression of the eGFP protein or by the resistance to puromycin. The insulators, placed on both sides of the expression cassette, ensured the expression of knock-in genes with the minimal impact on the neighboring genes. Finally, the LoxP sites allowed the conditional knock-in. The donor vector was created by cloning the left arm of the donor template sequence (containing the base substitution of rs4644, a portion of the exon 2 and the first 100 bases of the intron) in the MCS1, while the remaining intronic portion in the MCS2 (Fig. S4 panel A). Colony PCR and Sanger sequencing were used to identify the correct colonies.

### Isolation of gene-edited cells

Depending on the method of gene editing, we selected and isolated the mutated clones either by serial dilution/nested PCR (ssODN) or by FACS/antibiotics (HR vector).

(i) **Stringent dilution combined with nested PCR.** This method was employed for the cells gene edited by CRISPR/Cas9 vectors and ssODN. One microgram of each CRISPR/Cas9 vectors and 1 mM of ssODN were transfected by Lipofectamine 3000 in a six-well plate (about 250,000/well). Forty-eight hours after the transfection, cells were dissociated to single cells and seeded in p24 wells plate at 100 cells/well. At confluence, a part of cellular population was genotyped by nested PCR by the three silent mutations added into ssODN. The nested PCR product (227 bp) was a marker of the correct gene editing. All the primers are listed in Table S1.
(ii) **Transfections and screening of the gene-edited cells by antibiotics and FACS for standard and double nickase strategy**.
  This strategy was deeply discussed in the manuscript by Corrado et al. (2021). Briefly, 1 µg of CRISPR/Cas9 plasmids and donor vector was transfected into Nthy-Ori cells. Cells edited in the correct way showed both the puromycin resistance and GFP expression. For the isolation of the sub-population, cells were treated with puromycin (1 μg/ml) and ascertained by sorting-FACS for GFP marker cassette.
(iii) **Transfections and screening of the gene-edited cells by FACS for lentivirus strategy**.
  Nthy-Ori cells transduced with pCW-Cas9 and pLX-sgRNA vectors were cultured into a six-well plate (about 250,000/well) 1 day prior to transfection. One microgram of donor vector was transiently transfected using Lipofectamine 3000 according to the manufacturer's instructions. The treatment by doxycycline was performed at the same time of the cellular plating. For the selection of gene-edited cells we used FACS for GFP marker cassette.

### CRE recombinase excision of the selection cassette

As described previously, HR vector showed LoxP sites flanking the expression cassette of the two markers that can be removed by Cre recombinase activity (Fig. S4 panel B). Green and resistant puromycin cells were transfected by pPGK-Cre-bpA vector (Addgene plasmid # 11,543, kindly donated by the Klaus Rajewsky Lab) (Fig. S2 panel E). The selection of the positive cells (non-green) was performed by FACS using the green/puromycin resistant cells as control.

### Validation of the correct gene editing

For validation, DNA was extracted from gene-edited cells and a PCR encompassing the rs4644 polymorphism was carried out by using Q5^®^ Hot Start High-Fidelity DNA Polymerase (New England Biolabs, NEB, Ipswich, Massachusetts USA) followed by Sanger sequencing. Primer sequences are listed in Table S1.

### Tracking of insertions, deletions, and recombination (TIDER) analysis

TIDER was used for assessing the rate of HR events. It evaluates the frequency of small nucleotide changes introduced by CRISPR in association with HR and discriminates them against the background spectrum given by in/del mutations (Brinkman et al. 2018). The output consists of *R*^2^ as a measure of quality of the analysis (*R*^2^ = 1, maximal quality) and *P* value (*p*), as statistical significance.

## Results

**Fig. 1:**
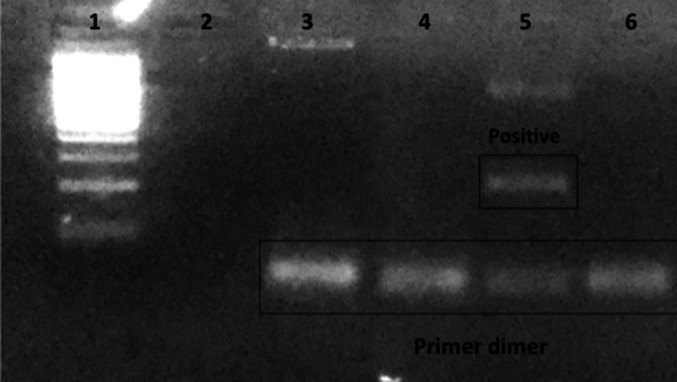
Nested PCR. Electrophoresis gel showing the nested PCR. Lane 1: 100-bp ladder, Lane 2: empty. Lanes 3–4: negative clones. Lane 5: 227 bp-long PCR product reporting a culture where the CRISPR/Cas9 + ssODN strategy worked. Lane 6: negative control

**Fig. 2:**
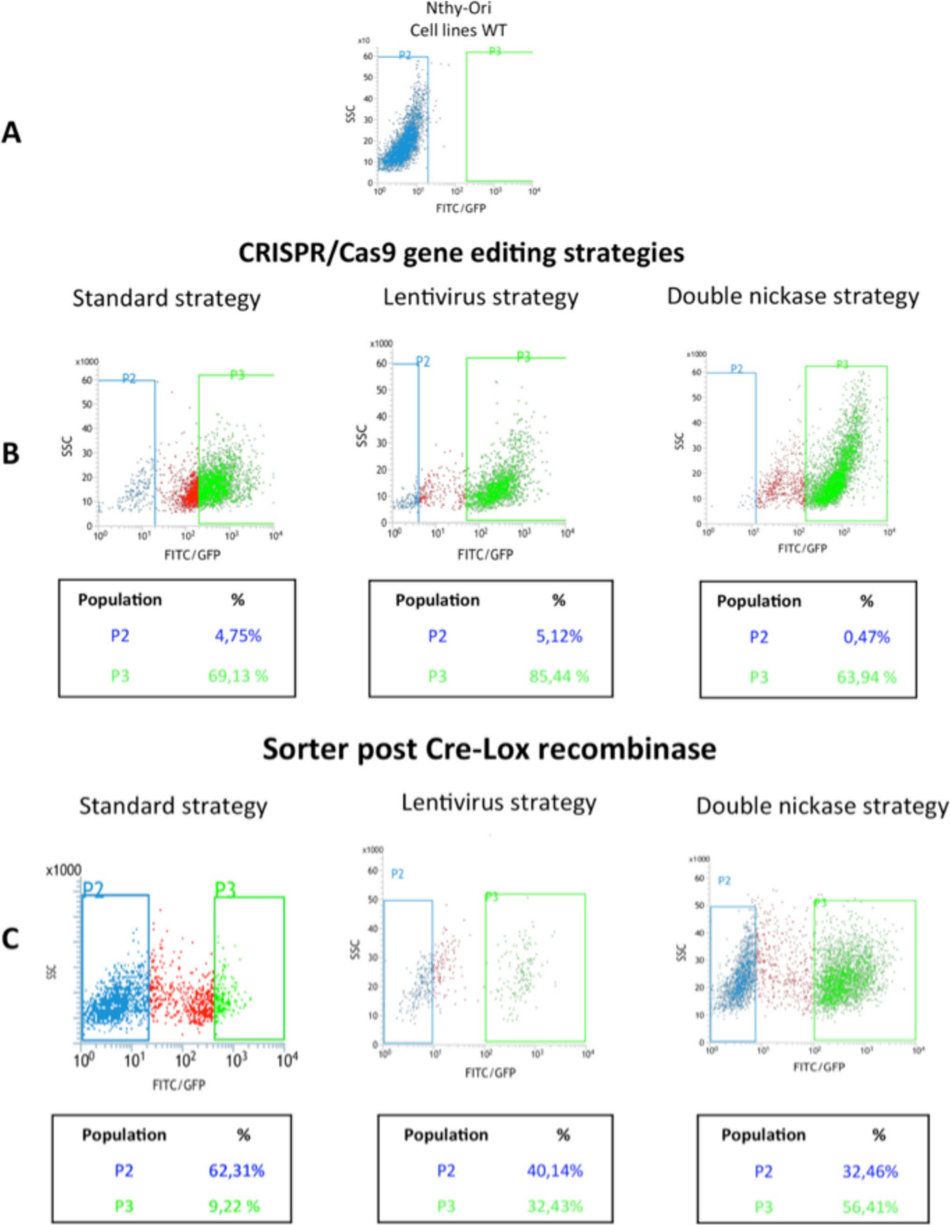
FACS outputs of Nthy-Ori cell lines. **A** Gating analysis of cells not undergone to gene editing to set the parameters. **B** FACS sorted cells undergone to different strategies of CRISPR/Cas9 gene editing (standard, with lentivirus, and with double nickase). Cells were sorted according to GFP expression (green area). **C** FACS sorted cells were Cre-loxed and subjected to a further FACS sorting for isolating those lacking the marker cassette (blue area)

**Fig. 3:**
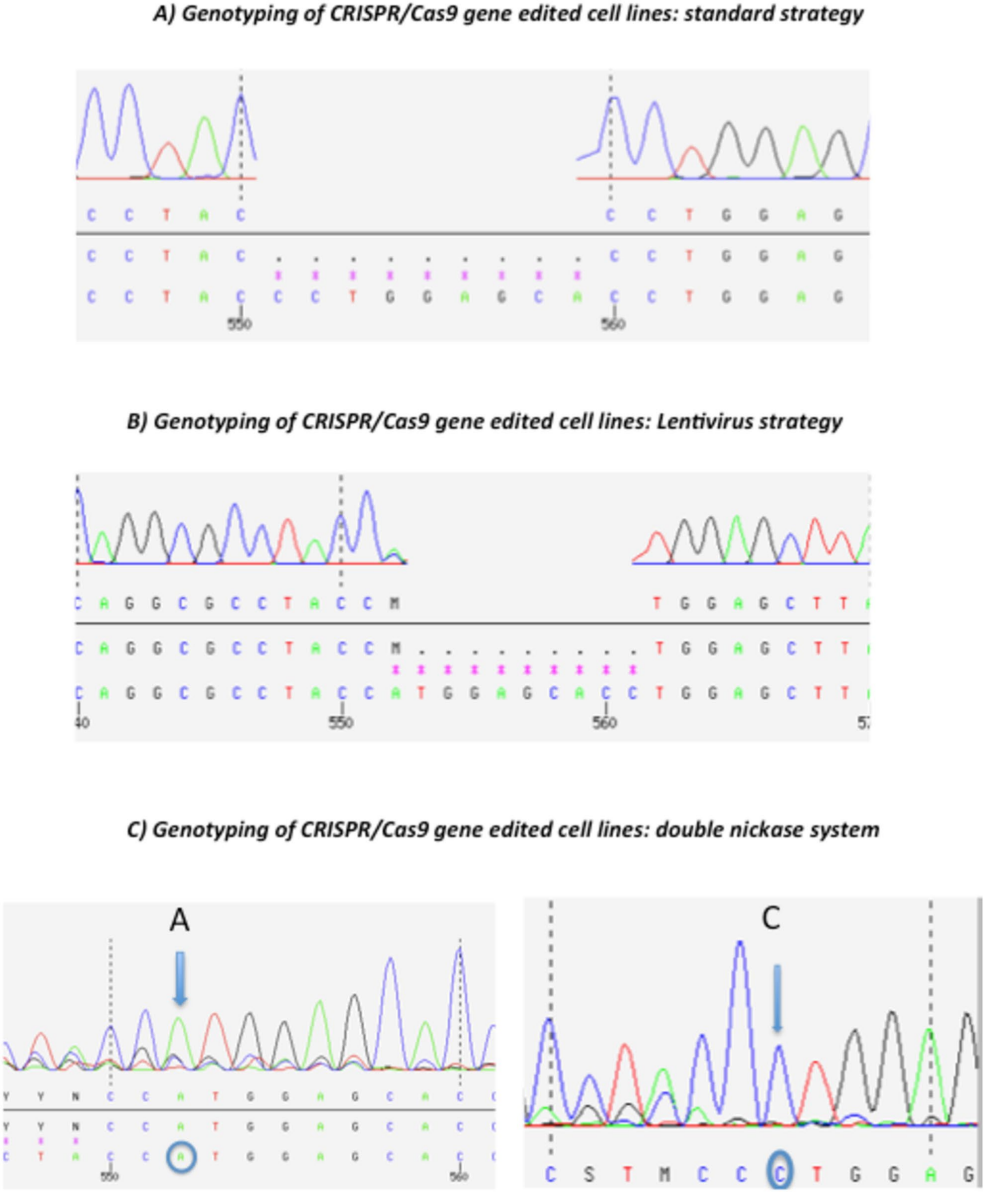
Evaluation of gene editing by Sanger sequencing. Chromatograms of the DNA region concerning the SNP rs4644 gene following CRISPR/Cas9 gene editing. Panels **A** and **B** report the 9-bp deletion following the application of the standard and lentivirus strategy. Panel **C** depicts the correct gene editing (circles indicate the gene-edited bases)

The chemical agents, Lipofectamine 3000 and Attractene, were tested to determine the agent for the delivery of the CRISPR/Cas9 system showing the highest transfection efficiency compatible with the least cell toxicity. After 48 h post-transfection of GFP expression vector (AAY-3), a gating FACS analysis revealed that Lipofectamine 3000 showed higher transfection efficiency (79.9%) than Attractene (39.8%) with comparable parameters of vitality, namely the shape and the size of the cells (21.7% and 29.9%, respectively) (Fig. S5). The gene-editing performed by the combo between CRISPR/Cas9 and ssODN was evaluated by nested PCR. The three inserted silent mutations were exploited for the design of insertion-specific primers. Thus, the locus was firstly amplified with general primers, then insert-specific primers were used to detect the presence of the variant DNA administered through the ssODN. The DNA was extracted from half of the cultures and subjected to the nested PCR. The 227 bp-long nested PCR product was detected only in the wells where at least one cell with the edited gene was present because the employed nested primers could specifically anneal to the target carrying the three silent mutations added in the ssODN (Fig. 1). The positive population was seeded once again at low concentration and the nested PCR was used again as a tool to verify that the insert was still present after several growth cycles. For cell lines co-transfected by CRISPR/Cas9 and HR vectors, puromycin and GFP markers were used in two consecutive steps. In the first step, cells were grown with puromycin allowing the selection of cells positively transfected. In the second step, once reached the confluence, cells were sorted depending on their expression of GFP, implying the selection of the cells with a stable incorporation of the donor DNA into their genome, as reported in Fig. 2. The three panels show the sorting of Nthy-Ori cells transfected with PX459 (original Cas9), double nickase system, and lentivirus. Later, the “green” subgroups were transfected by Cre-Lox vector to remove the markers cassette. Thus, cells were depleted by GFP and lacked the green fluorescence. Cells were FACS-sorted and those with the desired gene modification (i.e., edited at the polymorphic site, but lacking the marker cassette within the nearby intron) were collected as reported in the Fig. 2. The validation of gene editing was performed by Sanger sequencing that showed a 9–bps deletion in the gene-edited cells obtained by original and lentiviral systems, while a perfect knock-in was appreciable for the double nickase system (Fig. 3). Also the gene editing at mRNA level was evaluated only for the correct knocked-in Nthy-Ori cell lines. As reported by Corrado et al. (2021), the cDNA had the expected genotype and the single intronic LoxP (remaining following the Cre-Lox recombinase activity) did not affect the splicing process ending with a correct junction between exon II and exon III. The data were also corroborated by TIDER in silico analysis. TIDER estimates the frequency of designed (templated) small mutations in a pool of cells transfected with Cas9 + sgRNA + HR in terms of percentage of homology directed repair (HDR). It also determines the frequency of non-templated in/dels. The output revealed that the percentage of HDR was dependent upon the adopted CRISPR/Cas9 system, performing the knocking-in with a percentage that spanned from 36.8% (with *R*^2^ = 0.98) to 73% (with *R*^2^ = 0.99) (Fig. S6). It should be also noted that TIDER confirmed the 9-bp deletion detected by Sanger sequencing.

## Discussion

Nowadays, CRISPR/Cas9 is considered a very promising tool for gene editing. However, the system can present problems in its applicability due to, among the others, low transfection efficiency, toxicity, the impossibility to obtain pure clones of edited cells, and, last but not least, the off-target effects. In particular, this latter occurs because the PAM sequence (5´-NGG-3´) recurs frequently in the genome and there is tolerance of mismatches of the sgRNA (those at the 5´ site are more tolerated than that at the 3´ site where there is the “seed region”) (Jiang and Doudna 2015). In the last few years, several improvements have been suggested to overcome this problem, including the optimization of the sgRNA in silico tool design. Regarding the delivery system, in our cell model, the best results were obtained with Lipofectamine 3000 and the transduction with the lentivirus particles (pCW-Cas9 and pLX-sgRNA). Lipofectamine 3000 yielded a cellular toxicity similar to Attractene with a striking better transfection efficiency. Moreover, the use of the viral system simplified the forward steps. In fact, only the transduced cells could survive after the treatments with puromycin and blasticidin. The former allowed to select for the integration of the construct carrying the Cas9, the latter for the sgRNA. We also evaluated the standard and the double nickase system to create the DSB in the region adjacent to the targetable SNP and the latter one showed to be more effective. We obtained a successful gene editing both at genomic and at mRNA level as also reported in manuscript Corrado et al. (2021). We hypothesized that this result is correlated to the type of induced DNA breaks. In fact, the double nickase system creates SSBs that can be repaired in a more conservative manner than the DSB. Indeed, in the double nickase system the breaks are staggered and the presence of the “overhangs” could favor the DNA repair by HR whereas a blunt cut, typical of the standard strategy, could favor the NHEJ repair. Furthermore, as reported by Ma et al. (2014), the double nickase system enhances the specificity by avoiding the off-targets. Additionally, we noticed that the best sgRNA for gene editing was designed with the target site rs4644 in correspondence of the base before the PAM sequence (data not shown). However, we cannot affirm whether this could be a general rule for improving the efficiency of the sgRNA. We also tested two different donor DNAs, the ssODN or the HR vector. As proposed by Ran et al. (2013b), we designed an ssODN strand with three silent mutations both to mask other possible PAM sequences and to allow the detection and screening of the mutated cells by a nested and insertion-specific PCR. Theoretically, using ssODN, the gene-edited cell lines should have the same features of the parental ones with the only difference in the target allele. The limitation of this method is the difficulty to obtain pure clones. Cells seeded at low concentration lost the chemotaxis stimuli, very important for cellular proliferation in particular for non-malignant cells, as Nthy-Ori cells. With the HR vector we obtained the desired cells carrying either the A/A or C/C genotype at the rs4644 polymorphism in the most effective way because the presence of the marker cassette integrated within the intron II helped the selection of the modified cells. In this case, the cassette could be removed by the activity of Cre recombinase leaving only one LoxP site, that did not affect the splicing nor the mRNA expression of *LGALS3* (Corrado et al. 2021).

In summary, the standard CRISPR/Cas9 system is suitable for gene knock-out as it creates in/dels following the action of NHEJ, whereas it showed to be poorly effective for knocking-in the immortalized Nthy-Ori cell line. On the other hand, we showed that the combination of the double nickase system and HR vector was the most efficient method for knocking-in this cell line.

### Supplementary Information

Below is the link to the electronic supplementary material.

Supplementary file1 (TIFF 2927 KB)

Supplementary file2 (TIFF 13195 KB)

Supplementary file3 (DOCX 51 KB)

Supplementary file4 (TIFF 13195 KB)

Supplementary file5 (TIFF 1521 KB)

Supplementary file6 (TIFF 2702 KB)

Supplementary file7 (DOCX 95 KB)

Supplementary file8 (DOCX 21 KB)

## Abbreviations

CRISPR/Cas9: Clustered regularly interspaced short palindromic repeats/associated protein-9 nuclease
DSB: Double-strand DNA break
FBS: Fetal bovine serum
FACS: Fluorescent-activated cell sorter
HR: Homologous recombination
HDR: Homology directed repair
HEK: Human embryonic kidney
MCS: Multiple cloning sites
NHEJ: Non-homologous end joining
PAM: Protospacer adjacent motif
SNP: Single-nucleotide polymorphism
SSBs: Single-strand breaks
ssODN: Single-strand donor oligonucleotide
TIDER: Tracking of insertions deletions and recombination
