# Supplementary figures and images for: Strategies for single base gene editing in an immortalized human cell line by CRISPR/Cas9 technology
Source: 3 Biotech. 2024 Jan 19;14(2):45. doi: 10.1007/s13205-023-03878-4 (PMC10798938; doi:10.1007/s13205-023-03878-4)

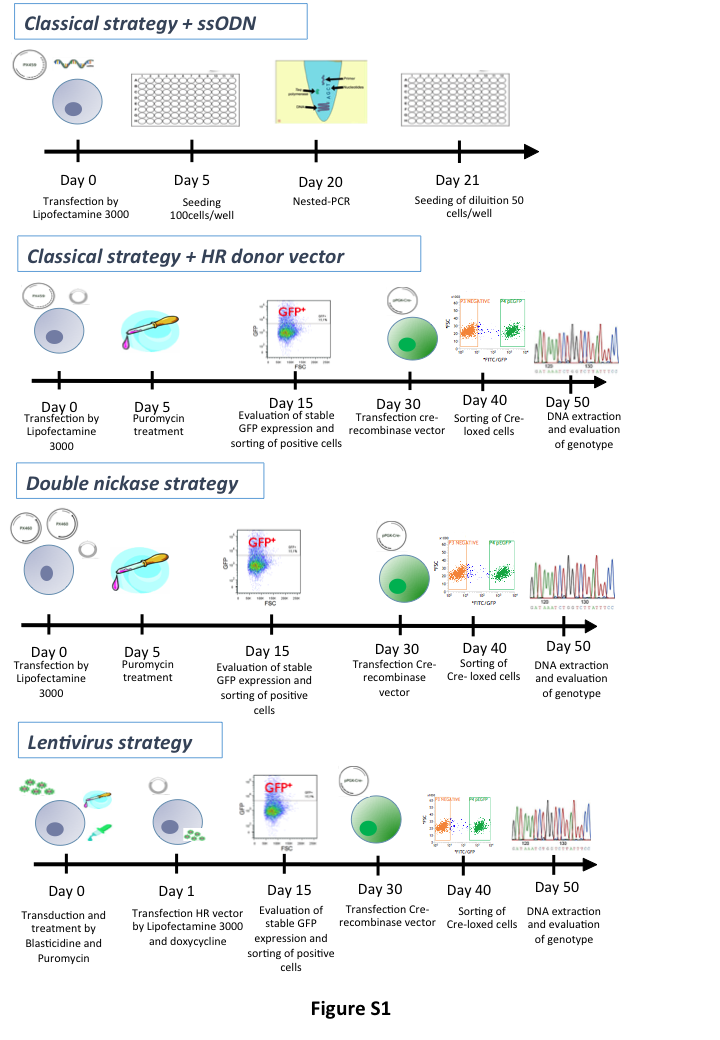

Supplement: Supplementary file 1 — Supplementary file1 (TIFF 2927 KB) [file 13205_2023_3878_MOESM1_ESM.tiff]

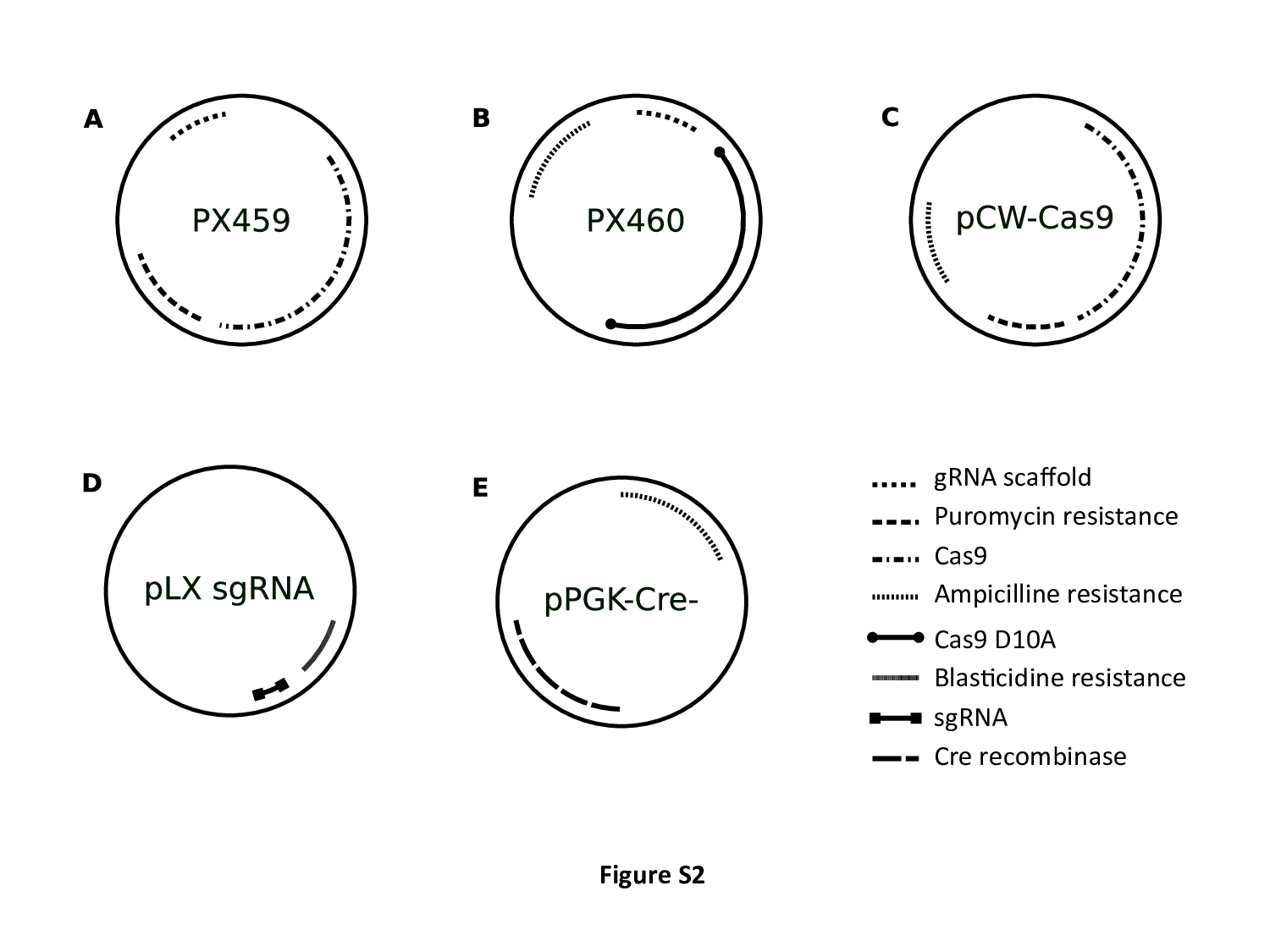

Supplement: Supplementary file 2 — Supplementary file2 (TIFF 13195 KB) [file 13205_2023_3878_MOESM2_ESM.tiff]

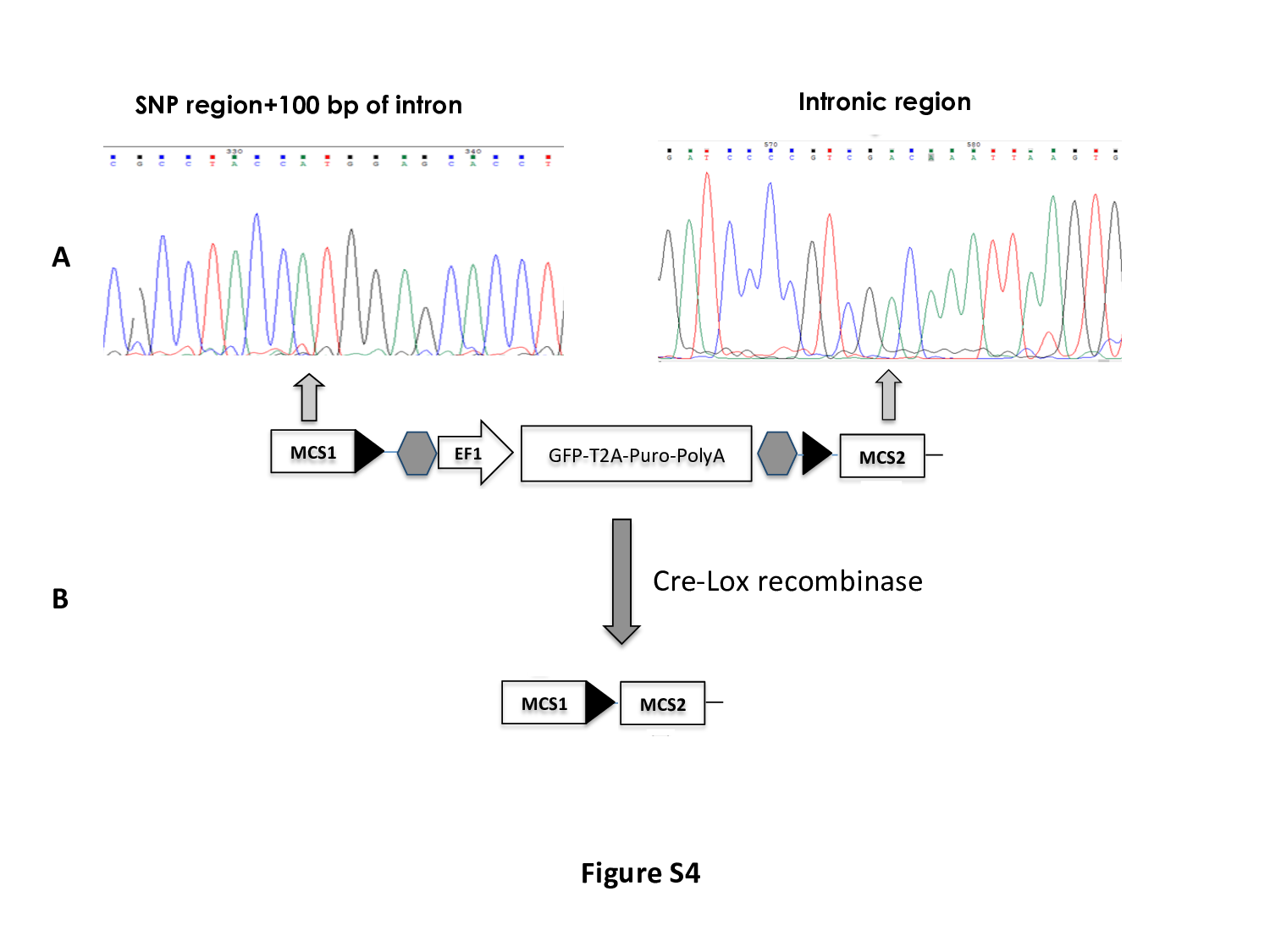

Supplement: Supplementary file 4 — Supplementary file4 (TIFF 13195 KB) [file 13205_2023_3878_MOESM4_ESM.tiff]

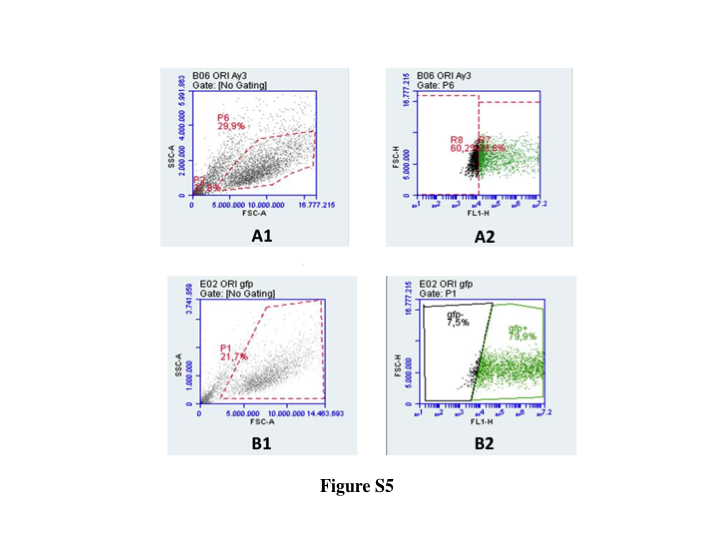

Supplement: Supplementary file 5 — Supplementary file5 (TIFF 1521 KB) [file 13205_2023_3878_MOESM5_ESM.tiff]

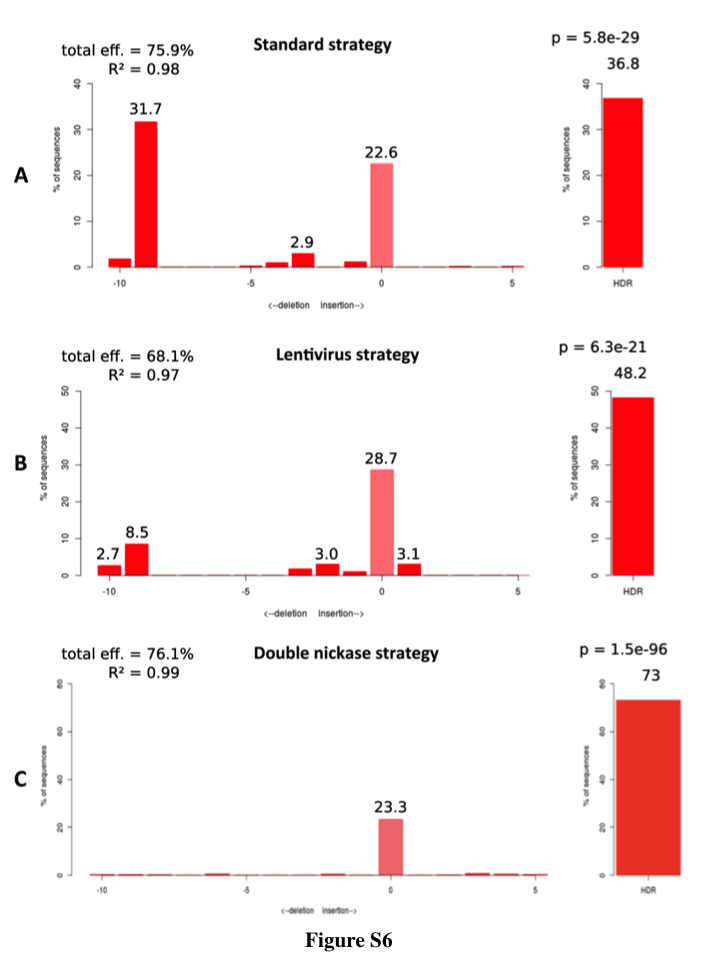

Supplement: Supplementary file 6 — Supplementary file6 (TIFF 2702 KB) [file 13205_2023_3878_MOESM6_ESM.tiff]
