# Supplementary material for: Strategies for single base gene editing in an immortalized human cell line by CRISPR/Cas9 technology
Source: 3 Biotech. 2024 Jan 19;14(2):45. doi: 10.1007/s13205-023-03878-4 (PMC10798938; doi:10.1007/s13205-023-03878-4)
Supplement: Supplementary file 3 — Supplementary file3 (DOCX 51 KB) [file 13205_2023_3878_MOESM3_ESM.docx]

| CtgtcccgtaattgtgtatgtctttctttccagCTCCATGATGCGTTATCTGGGTCTGG  >>>>>>>>>>>>>>>>>>>>>> ->->->->->->->->->->->->->->  AAACCCAAACCCTCAAGGATGGCCTGGCGCATGGGGGAACCAGCCTGCTGGGGCAGGGGGCTA  CCCAGGGGCTTCCTATCCTGGGGCCTACCCCGGGCAGGCACCCCCAGGGGCT(T/C)ATCCTGG  ACAGGCACCTCCAGGCGCCTACC**(C/A)**TGG**T**GC**T**CC**C**GGAGCTTATCC(C/T)GGAGCACCT  <<<<<<<<<<<<<<<<<<<  GCACCTGGAGTCTACCCAGGGCCACCCAGCGGCCCTGGGGCCTACCCATCTTCTGGACAGCCAAGTGCC(A/C)CCGGAGCCTACCCTGCCACTGGCCCCTATGGCGCCCCTGCTGGGCCACTG  <-<-<-<-<-<-<-<-<-<-<-<-<- | **rs4644; (Pro 64 His)**; |
| --- | --- |

**Figure S3**
