# Supplementary material for: Strategies for single base gene editing in an immortalized human cell line by CRISPR/Cas9 technology
Source: 3 Biotech. 2024 Jan 19;14(2):45. doi: 10.1007/s13205-023-03878-4 (PMC10798938; doi:10.1007/s13205-023-03878-4)
Supplement: Supplementary file 7 — Supplementary file7 (DOCX 95 KB) [file 13205_2023_3878_MOESM7_ESM.docx]

**Supplementary Figure Legend**

**Figure S1 Scheme of the strategies.**

Timeline overview of the all strategies adopted to perform a single base gene editing of SNP rs4644 within *LGALS3* in the Nthy-Ori cell line.

**Figure S2 Graphical structures of all vectors employed.**

A) All in one Cas9 vector used in the standard strategy. B) All in one Cas9 mutated vector employed in the double nickase system. C) Cas9 expression vector and D) vector for cloning the sgRNA both used for lentivirus approach. E) Cre-Lox recombinase vector.

**Figure S3 Features of ssODN.**

At 40th position is located the SNP rs4644. The three bold bases after SNP indicated the silent mutations inserted to mask other PAM sequences and useful for the selection of the correct gene edited cells by nested-PCR screening.

**Figure S4 Scheme of donor vector (HR) pre and post Cre-Lox recombinase.**

Panel A depicts the region encompassing the SNP rs4644 and the first 100 bp of the nearest intron cloned in the multiple cloning site (MCS) 1 and the remain portion of the intron cloned in MCS2. The triangles show the LoxP sites located aside the marker cassette and the hexagons show the insulators. After the Cre-Lox recombinase (panel B), adopted to remove the marker cassette post gene editing and cell sorting, single LoxP site is still present but it doesn’t affect the splicing mechanism.

**Figure S5 Evaluation of chemical agents.**

Comparison between Attractene (panel A) and Lipofectamine 3000 (panel B) for transfection efficiency of AAY-3 vector in Nthy-Ori cell lines measured by FACS analysis. Figures A1 and B1 show the cells population undergone to gating analysis. Panel 2 (A2 and B2) reports the gating analysis, where the green area shows the cells transfected and expressing the GFP.

**Figure S6 TIDER analysis.**

Comparison of TIDER analysis between the 3 gene editing strategies: (A) standard, (B) lentivirus, and (C) double nickase. Panel C shows the double-nickase strategy having the highest percent of HR, whereas panels A and B report a reduced rate of HR accompanied by an important share of ins/del likely related to NHEJ activity.
