# Supplementary material for: Strategies for single base gene editing in an immortalized human cell line by CRISPR/Cas9 technology
Source: 3 Biotech. 2024 Jan 19;14(2):45. doi: 10.1007/s13205-023-03878-4 (PMC10798938; doi:10.1007/s13205-023-03878-4)
Supplement: Supplementary file 8 — Supplementary file8 (DOCX 21 KB) [file 13205_2023_3878_MOESM8_ESM.docx]

**Supplementary Table S1.** Primers list

| ***Application*** | ***Primer Sequence*** |
| --- | --- |
| *MCS1 region* | F: 5’AGTGAATTCGAGCTCGGTACCCTGTCCCGTAATTGTGTATGTC-3’ |
|  | R: 5’-CTGATGCATTCGCGAGGTACCTTTGACTCTACCAACATACACC-3’ |
| *MCS2 region* | F: 5’-CTAGATCGGATCCCCGTCGACAAATTAAGTGTTCATATTGAG-3’ |
|  | R: 5’-AAGCTTGCATGCAGTCGACGTGAAAATTCAACCCTTAG-3’ |
| *Validation of cloning MCS1* | F: 5’-AACTGTTGGGAAGGGCGATC-3’ |
|  | R: 5’-TAGCGGGGGAGGGACGTAATT-3’ |
| *Validation of cloning MCS2* | F: 5’-GACGTAATTACATCCCTGGG-3’ |
|  | R: 5’-GCAGCGAGTCAGTGAGCG-3’ |
| *sgRNA_1 (for C allele)* | F: 5’-CACCGGGCGCCTACCCTGGAGCACC-3’ |
|  | R: 5’-AAACGGTGCTCCAGGGTAGGCGCCC-3’ |
| *sgRNA_2 (negative strand)* | F: 5’- CACCGGCCTGTCCAGGATAAGCCCC -3’ |
|  | R: 5’- AAACGGGGCTTATCCTGGACAGGCC-3’ |
| *sgRNA_3 (for A Allele)* | F: 5’- CACCGGCACCTCCAGGCGCCTACCA-3’ |
|  | R: 5’- AAACTGGTAGGCGCCTGGAGGTGCC-3’ |
| *Colony PCR for sgRNA_1* | F: 5’-CGATACAAGGCTGTTAGAGAG-3’ |
|  | R: 5’-CTAAAACGGTGCTCCAGG-3’ |
| *Colony PCR for sgRNA_2* | F: 5’-CGATACAAGGCTGTTAGAGAG-3’ |
|  | R: 5’- CTAAAACGGGGCTTATCC-3’ |
| *Colony PCR for sgRNA_3* | F: 5’-CGATACAAGGCTGTTAGAGAG-3’ |
|  | R: 5’- CTAAAACTGGTAGGCGCCT -3’ |
| *Evaluation of integration of MCS1* | F: 5’-GAGAAATCCTCTGAGTAGCGGG-3’ |
|  | R: 5’-ATGTTTTGAGTGGAAAGATCTGATG-3’ |
| *Evaluation of integration of MCS2* | F: 5’-GCTATCTGGTCTCCCTTCCG-3’ |
|  | R: 5’-GTGCTGTCTTCTGCATAGTCCTG-3’ |
| *Validation of gene editing at DNA level* | F: 5’-CTGTCCCGTAATTGTGTATGTC-3’  R: 5’-ACTCTACCAACATACACCCAT-3’ |
| *Validation of gene editing at cDNA level* | F: 5’-TGCGTTATCTGGGTCTGGAA-3’  R: 5’-ATGAAGCACTGGTGAGGTCT-3’ |
| *Pre-Nested PCR* | F: 5’-CTCCATGATGCGTTATCTGGGTCTGG-3’  R: 5’-CAGTGGCCCAGCAGGGGCGCCATAGG-3’ |
| *nested PCR* | F: 5’-CTGTCCCGTAATTGTGTATGTC-3’  R: 5’-GGATAAGCTCCGGGAGCA-3’ |
| *pLXsgRNA_1_Overlap_PCR* | F: 5’-AAACTCGAGTGTACAAAAAAGCAGGCTTTAAAG-3’  R: 5’-GGTGCTCCAGGGTAGGCGCCGGTGTTTCGTCCTTTCC-3’ |
| *pLXsgRNA_2_ Overlap_PCR* | F: 5’-GGCGCCTACCCTGGAGCACCGTTTTAGAGCTAGAAATAGCAA-3’  R: 5’-AAAGCTAGCTAATGCCAACTTTGTACAAGAAAGCTG-3’ |
| *TBP_Housekeeping gene* | F: 5'-GCGGTTTGCTGCGGTAATC-3'  R: 5'-TCTGGACTGTTCTTCACTCTTGG-3' |
| *HPRT1_Housekeeping gene* | F: 5'-GATGGTCAAGGTCGCAAG-3'  R: 5'-GGGCATATCCTACAACAAACTT-3' |
| *RPLP0_Housekeeping gene* | F: 5'-CCTCATATCCGGGGGAATGTG-3'  R: 5'-GCAGCAGCTGGCACCTTATTG-3' |
| *Cas9* | F: 5’-GTACCCCACCATCTACCACC-3’  R: 5’-GGATGAACAGCTTGTCCACG-3’ |
